# President Roosevelt and the Tuberculosis Congress

**Published:** 1906-11

**Authors:** 


President Roosevelt and the Tuberculosis Congress.—He sends assurances of his interest in the meeting, and best wishes for its success.

American International Congress on Tuberculosis, New York City, November 14, 15, and 16, 1906.

OFFICE OF THE PRESIDENT.

Austin, Texas, October 22, 1906.

*Hon. Theodore Roosevelt, President of the United States of America, Washington, D. C.*

Mr. President: On behalf of the management we have the honor to inform you that the fourth biennial session of the American International Congress on Tuberculosis will be held in New York City November 14th, 15th, and 16th (prox.), and to invite you to be present.

You are doubtless informed as to the magnitude of the evil which this Congress is earnestly seeking to devise and recommend means to combat. We recall with satisfaction your active and efficient work in the cause when Governor of New York, and more recently in ordering the enforcement of sanitary precautions in all the departments at Washington; but it may not be known to you that while yellow fever, according to the United States statistics, during a century, has caused an average of one thousand deaths in America in a year, consumption destroys one thousand lives every two and a half days. It would seem that in view of this appalling fact no argument should be necessary to convince those who have the making of the laws that prompt and effectual action should be at once instituted for the arrest of this destructive disease. It is largely preventable; but to even limit the spread requires authority of law, co-operation, efficient action, and ample means. Practically nothing is being done by the general government to reduce the mortality of consumption in this country.

We are encouraged to hope that Your Excellency will lend to the movement the great weight and influence of your presence and cordial support, at least at the opening of the Congress. It would give an impetus to the work, and redound to the further glory of this great Republic, a Republic whose greatness has been so notably enhanced by your own wise administration. America, foremost in so many reforms and advances, should not be second in sanitary reform for the benefit of the public health. So devastating a disease is doubtless a factor in crime, prostitution and pauperism, and legislation in the interest of the public health is legislation in the interest of public morals. The world is aroused to the necessity of action looking to the control of tuberculosis, and we note in Germany, France, and England there has already been a marked decrease in the mortality rate. His Majesty the King of England, in June personally opened the great Charity Sanitarium for Consumptives at Sussex, an institution endowed by Sir Ernest Cassell with $1,000,000.

The management have the assurance of a large and enthusiastic attendance, not only from the North American and South American States, but from Europe, in response to the invitation sent out by the Hon. Elihu Root, Secretary of State, to all foreign countries with which we have relations through our diplomatic service.

May we not hope that Your Excellency will take an active interest in the great humanitarian work the Congress is seeking to do, and honor us with your presence, if only for a few minutes at the opening of the Congress?

We have the honor to be, Mr. President,

Yours very respectfully,

F. E. Daniel, President,

M. M. Smith, Secretary,

American International Congress on Tuberculosis, 1906.

THE WHITE HOUSE,
WASHINGTON.

October 27, 1906.

*Dr. F. E. Daniel, President, American International Congress on Tuberculosis, Austin, Texas.*

MY DEAR SIR: The President thanks you cordially for your kind letter of the 23d instant inviting him to attend the fourth biennial session of the American International Congress on Tuberculosis. He regrets, however, that it will be impossible to accept, as he will be on his Panama trip on the dates mentioned.

Assuring you of the President's interest in the work of your Congress, and conveying his good wishes for the success of the coming session, I am, Very truly yours,

WM. LOEB,
Secretary to the President.

---

LETTER FROM THE GOVERNOR OF TEXAS.

EXECUTIVE OFFICE,
STATE OF TEXAS, AUSTIN.

November 1, 1906.

*Dr. F. E. Daniel, President American International Congress on Tuberculosis, Austin, Texas.*

MY DEAR SIR: I have the honor to acknowledge the receipt of your special invitation to attend the American International Congress on Tuberculosis in New York on the 14th, 15th, and 16th inst. I regret exceedingly that my official duties and engagements will prevent me from complying with your courteous request. I regard this meeting of the medical profession as being one of the most important that has ever been held. I feel a deep interest in its success and have no doubt that much good will be accomplished thereby in the alleviation of this trouble which affects so many people.

With best wishes for the success of your Congress, I remain,

Very respectfully,

S. W. T. LANHAM,
Governor.

---

IMPORTANT NOTICE.—Delegates to the American International Congress on Tuberculosis and ladies accompanying them, are entitled to return on a one-third fare rate. See editorial. Physicians, other than delegates, who desire to go to New York at that time, can have the benefit of the same rate (one-third fare) by registering with the committee at the Astor House and becoming members of the Congress by the payment of the enrolling membership fee of $1. The time for return is not limited, I think.

---

The Congress will be held in the assembly hall of the Astor, which, I understand, has been placed at the service of the Congress, complimentary.

---

The President and the Secretary of the Congress will pass through Texarkana Saturday, November 10th, and through St. Louis — over the Iron Mountain route — early Monday morning, November 12; thence to Buffalo over the Wabash system, and they would be glad to have delegates and others join them, so as to make a party and secure a special through sleeper. Correspond with Secretary Smith, meantime, and arrange to meet us.

